# Communicating the consensus on climate change to college biology majors: The importance of preaching to the choir

**DOI:** 10.1002/ece3.5960

**Published:** 2020-01-09

**Authors:** Jeremy D. Sloane, Jason R. Wiles

**Affiliations:** ^1^ Center for Teaching Excellence University of Virginia Charlottesville Virginia; ^2^ Department of Biology Syracuse University Syracuse New York; ^3^ Department of Science Teaching Syracuse University Syracuse New York

**Keywords:** climate change, consensus messaging, Gateway Belief Model, scientific consensus, undergraduate education

## Abstract

College and university biology majors who are not climate change deniers may yet be unaware of the degree of scientific consensus on climate change and unprepared to communicate about climate science to others. This study reports on a population of climate change accepting biology majors at a large, private research university in the American northeast. Our students tended to greatly underestimate the degree of scientific consensus around climate change, to be only moderately worried about climate change, and to be unconfident in their ability to communicate about the state of the scientific consensus around climate change. After an introduction to the scholarly literature that substantiates and quantifies the scientific consensus on climate change in the context of a course on biological research literature, our students showed significant increases in their estimates of the consensus on climate change, and their estimates were more accurate. Additionally, they became more worried about climate change as well as more confident in their ability to communicate about the scientific consensus to others. These results are in line with the Gateway Belief Model, which positions perception of scientific agreement on climate change as an important driver of acceptance and motivation toward action.

## Graphical abstract

Upper‐division biology majors at a research university who were not climate change deniers were found to greatly underestimate the scientific consensus on climate change, to be only moderately worried about climate change, and to have little confidence in communicating to others about the level of scientific agreement climate change. After exposure to scholarly literature establishing and quantifying the consensus, students were significantly more accurate in their estimates of scientific consensus on climate change, more worried about climate change, and more confident in their ability to communicate about the consensus to others.

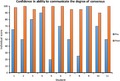

## 1 INTRODUCTION

**Figure 1:**
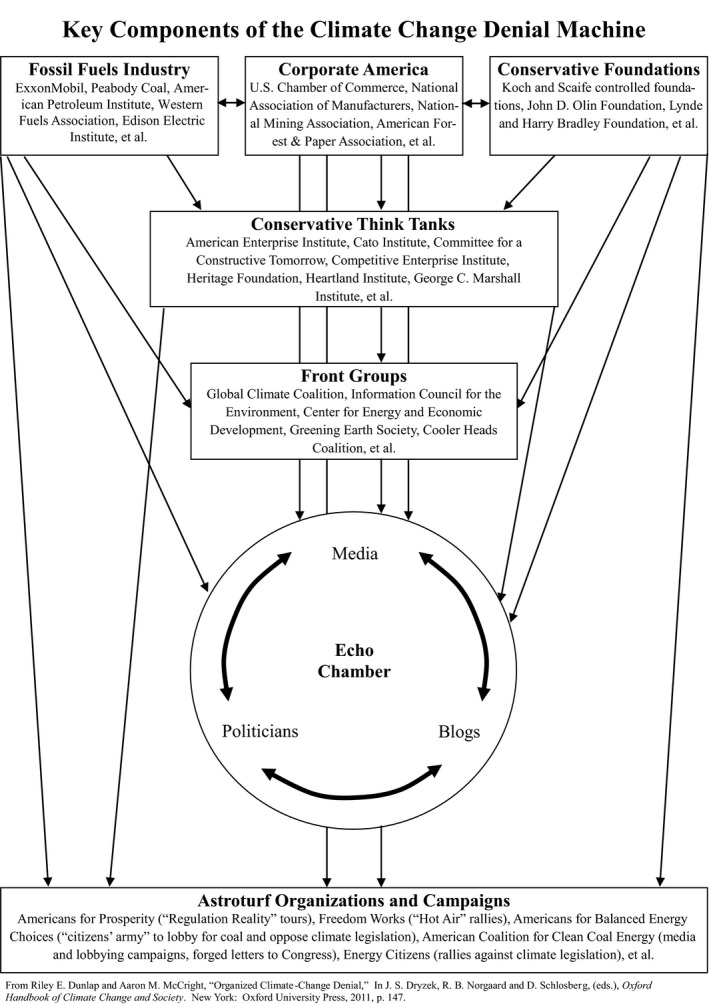
Key components of the climate change denial machine. From Dunlap and McCright (2011)

Perhaps especially in the United States, the lack of understanding of and concern about climate change among the general public is a direct result of concerted efforts to undermine climate science, or what might be aptly described as the “climate change denial machine.” (Dunlap & McCright, 2011, 2015) (see Figure 1). This complex construct serves to manufacture and amplify doubt regarding the veracity of the scientific consensus on human‐induced climate change in order to undermine support for climate policy. Each of the cogs of the Climate Change Denial Machine—the fossil fuel industry, Corporate America, conservative foundations, conservative think tanks, front groups, and pseudograssroots or “astroturf” organizations and campaigns—has targeted political conservatives based on the notion that they are predisposed to be skeptical of anything that raises the specter of governmental regulation. The idea is to keep these conservatives in the echo chamber (consisting of media, politicians, and blogs), which reinforces climate change denialism even as it shields deniers from counter information.

The machine is apparently quite effective at keeping climate change deniers within their insular bubbles. Fewer than half of Americans report hearing about global warming in the media at least once per month, and only a quarter of the population say they hear people they know discussing global warming at least monthly (Leiserowitz, Maibach, Roser‐Reouf, Rosenthal, & Cutler, 2017). The result is that, despite the near‐unanimous consensus among the world's experts in climate science and the potentially devastating consequences of climate change, understanding and concern among the American public are troublingly low. Only just over half of Americans are worried about global warming, and only about one in eight Americans understands that almost all climate scientists (above 90%) agree that anthropogenic global warming is happening (Leiserowitz et al., 2017; ). This last statistic is particularly troublesome, as perception of scientific consensus appears to be a “gateway belief” to acceptance, support for action and climate policy, and injunctive beliefs (or beliefs that certain individuals and entities should be doing more to address global warming) (Ding, Maibach, Zhao, Roser‐Renouf, & Leiserowitz, 2011; Linden, Leiserowitz, Feinberg, & Maibach, 2015; van der Linden, Leiserowitz, & Maibach, 2019; McCright, Dunlap, & Chenyang, 2013).

The halls of academia, perhaps particularly those in biology departments, have become echo chambers as well, though the comfortable assumption here is that we are all on board with the science on climate change. Much as with evolution and other areas of well‐established scientific consensus, biology faculty may teach about climate change from the perspective of knowing the strength of the consensus—without considering that students in the life sciences, especially undergraduates, probably do not, and they may not understand how such a consensus might be reached or measured.

Climate denial may not be as rampant on our campuses as it is among the general public, especially among students of the life sciences, but we must guard against merely assuming that our students are attitudinally aligned with the consensus on climate change. If we want to prepare our students, many of whom return to home communities rather removed from our ivory towers, to engage a broader, nonscientist public; we must ensure that they also understand the source and magnitude of the consensus and are competent and confident in communicating it to others.

## 2 OUR EXPERIENCE WITH STUDENTS IN AN INTRODUCTION TO RESEARCH LITERATURE COURSE

In the context of a broader, IRB‐approved study of the potential impacts of undergraduate biology students engaging with scientific literature, we had an opportunity to measure how learning about the scientific consensus around climate change might influence students' understandings of the consensus, their level of worry about climate change, and their confidence in their ability to communicate about it to others. The course itself was similar to other iterations of the same course designed to introduce students to research in our Biology Department (Carter & Wiles, 2017; Schmid & Wiles, 2019) and grounded in the prior research indicating that using scholarly literature in undergraduate science courses is a particularly effective way to improve students' scientific content knowledge in the specific area of study (Carter & Wiles, 2017; DebBurman, 2002; Hoskins & Kenyon, 2014; Kozeracki, Carey, Colicelli, & Levis‐Fitzgerald, 2006; Schmid & Wiles, 2019; Yeong, 2015).

Ten out of the eleven students who participated in this course were seeking degrees in either biology or biochemistry, while the remaining student had not yet declared an academic major. The majority of them (seven of the eleven) were sophomores, two were juniors, and two were seniors. Nine of the students were United States citizens while two were international students. All of our students indicated at the beginning of the semester that they were interested in joining a research laboratory as an undergraduate, yet less than a third of them reported that they had ever previously read or discussed primary research literature in another college science course.

As one of the secondary goals of the course was to introduce students to the work of faculty members with whom they might engage in undergraduate research, this one‐semester, two‐credit course involved students reading, discussing, and writing about papers from research laboratories in our Biology Department. Prior to reading articles from our faculty research laboratories, the students were introduced to different kinds of scholarly literature, such as primary research reports, methods papers, systematic reviews, and meta‐analyses, through instructor‐selected examples of each. During this portion of the course, students were assigned to read and construct written responses to two climate change consensus papers—one that quantified the level of consensus on human causation via meta‐analysis (Cook et al., 2013) and one that reviewed several different consensus estimates (Cook et al., 2016). For these papers, as well as other papers assigned in the course, students were required to complete a Figure Facts template (Round & Campbell, 2013) prompting them to take a data‐centric approach to reading the papers and to post a response to the papers in an online discussion. The students were asked to reply to at least two other students' responses. Only students participated in the online discussions, and the instructor facilitated in‐class discussion of the assigned articles. The remainder of the course contained no further presentation, activities, nor assigned readings directly related to climate change. Typical exchanges between students in the online discussion included:

**Student's reflective comment:**

In Cook 2016, I found it most alarming how high consensus numbers among experts compared to how the general public feels about the subject. Specifically, the statistic that a mere 12% of the American population accurately estimate that 91%–100% of scientists are in accord baffled me. As I reflected, I would have guessed that 75% of Americans support anthropogenic global warming. Reading further, the cause is said to be rooted in our early education, with middle and high school teachers instilling doubt in their pupils. I began to think of my AP environmental science class, and remembered learning more about policy on climate change rather than the overwhelming consensus. It seemed more like we students were forming an opinion on climate change rather than being lectured on it, focusing more on how humans are trying to alleviate greenhouse gases rather than on expert opinions and primary literature. My curiosity leads me to wonder if aggressive installation of the scientific consensus into public curriculum would increase the statistic from 12%.

**Typical students' responses to this comment:**

- I also remember having classes where, instead of being given the overwhelming scientific consensus on AGW, students have been taught to form an opinion on climate change. Rather than treating it as the scientific issue that it is, people treat it as a political position. Students should be taught how climate change could affect our world and what we can do.
- I agree that students are being allowed to make an opinion on climate change instead of teaching us on the cause of climate change which has been evident since 1990. It makes me wonder what are the reasons for not showing the truth of AGW instead of portraying how humans are supposedly trying to end global warming.
- I agree that students generate opinions on global warming in secondary school instead of being taught the direct facts and statistics that scientists have found supporting the causes of global warming. I have always found issue with this because I remember in high school I talked more about global warming in my economics/government class in response to how climate change and global warming affects supply and demand or which House representative supported certain global warming policies, while we barely touched the subject of global warming in my science courses.

**Student's reflective comment:**

The discussion of human influence on global climate change has been greatly influencing politics for much of the last 20 years. What I found most interesting about this research study is how the author, through many massive surveys and collections of articles and abstracts already in existence, proves the public belief that climate change is widely disputed among scientists to be unfounded. Figured 1 and 2 of the research paper show that there has been no increase in rejections of AGW since 1990 where it sat at 0.7% of abstracts analyzed. The media seems to portray this research in a different light, as up until this point I did not realize how many climate experts proved human influence on climate change in their research (as shown in Figure 1 of the review). The public is being largely influence by media distortion of this data and Cook shows the research that the consensus on AGW has been in existence since 1990.

**Figure 2:**
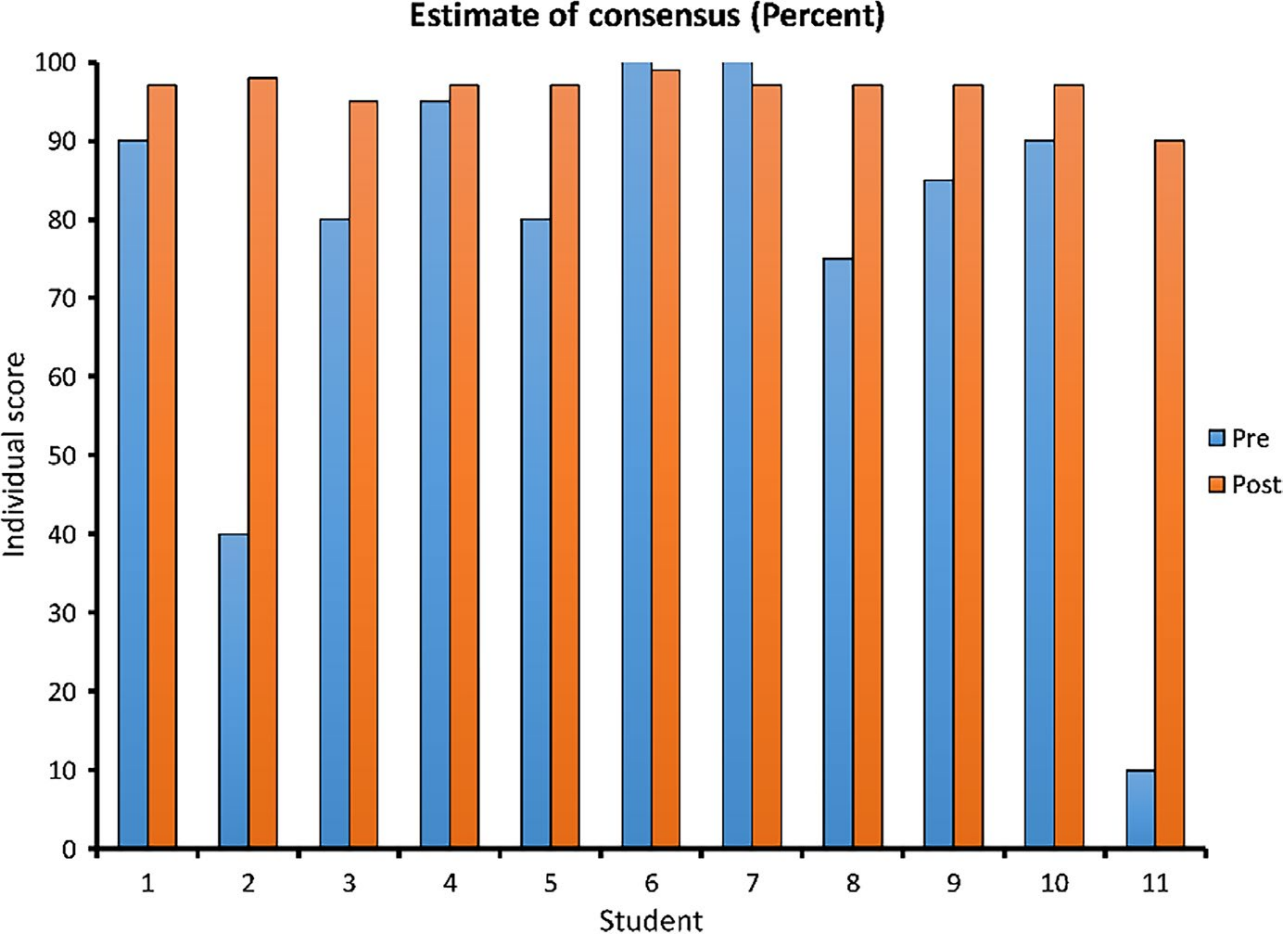
Perceived consensus. Estimates of the percent consensus before and after reading and discussion of a meta‐analysis and a systematic review of current climate science articles. A Wilcoxon signed‐rank test revealed a significant increase in participants' perceptions of scientific consensus on climate change (*Z* = −2.580, *p* = .010)

**Typical students' responses to this comment:**

- I agree that the media has changed the perception of global warming, not emphasizing the fact that climate scientists agree that humans are the cause of recent global warming. Although media has not shown this research in the right light yet, in the future it can possibly be used to show the negative impact humans have been having on climate change and the agreement of experts on AGW.
- Ultimately, I find it unjust that human beings are being robbed of the truth that we are changing the planet for the worse, and that nearly 100% of all experts agree on this. Yet hard cold facts can be muddied with personal incentive, which worries me to wonder what else the general public is in the dark about.
- I definitely agree that the media distorts the data the climate scientists have proven. We know that the general public has access to these AGW articles, but I do not think they necessarily understand what the paper or abstract is saying. That, I think, is where the media comes in. The media doesn't cover scientists/researchers, just people saying what the people want to hear. So when people are only listening to what the media has to say, they do not understand or know the consensus of these climate scientists and how we are being affected by AGW.

Prior to these assigned readings and online discussions, a portion of the first class period of the semester was spent collecting initial data including participants' estimates of the degree of scientific consensus around climate change, their level of worry about climate change, and their confidence in their ability to communicate the degree of scientific consensus to others. Readings including the two climate change consensus articles were assigned at the end of the first class to be read the following week. The second class session included in‐class small and large group discussions of the climate change consensus papers. Participants responded to the quantitative postexperience survey questions and qualitative climate change prompt online between the second and third classes. All data, including qualitative data gathered through students' online discussions, were collected through the Blackboard course management system. All students voluntarily participated in all data collection activities according to the approved protocol.

Given that these students had voluntarily enrolled in a course on scientific research literature at a large, private, research‐intensive (Carnegie R1 designated) university in the northeastern region of the United States, we did not expect our student population to be in denial regarding climate change to any large extent. And, indeed, there was little, if any, doubt among our students regarding the veracity of anthropogenic climate change, as measured by the instrument developed by van der Linden et al. (2015). However, prior to their experiences in this course, our students greatly underestimated the degree of the scientific consensus, expressed as a percent, which has been reported at 97.1% by Cook et al. (2013) in the most recent and rigorous meta‐analysis of human‐induced climate change among almost 12,000 peer‐reviewed climate change papers. (It should be noted that several surveys of climate scientists have revealed similar levels of consensus (Bray & Storch, 2007; Carlton, Perry‐Hill, Huber, & Prokopy, 2015; Doran & Zimmerman, 2009; Farnsworth & Lichter, 2012; Stenhouse et al., 2014; Verheggen et al., 2014)).

On the first day of our course, students were asked to respond to the following question: “To the best of your knowledge, what percentage of climate scientists have concluded that human‐caused climate change is happening? Answer between 0% and 100%” (van der Linden et al., 2015). Although none of them were climate change deniers, our students' estimates of the consensus within the climate science community returned a mean of only 76.8%, with estimates varying wildly (the standard deviation was 27.68). Values ranged from 10% to 100%. After reading and discussing scholarly literature regarding the consensus and how it is measured, the mean student estimate of the consensus for the post‐test was 96.45%. Values ranged from 90% to 99%. The median and mode were both 97%, accurately reflecting the actual degree of consensus, and the standard deviation was only 2.34. A Wilcoxon signed‐rank test was conducted to determine whether there was a significant difference in consensus estimates between the pretest and post‐test. Results indicated that consensus estimates were significantly higher in the post‐test than pretest (*Z* = −2.580, *p* = .010; Figure 2), and they were closer to the actual level of consensus.

When participants were asked via online discussion prompts whether their experiences in the course had influenced their understanding of the degree of scientific consensus on human‐caused climate change, 82% (9) answered that it had. Representative quotes included:

“Yes it has. Meta‐analysis of the scientific community's consensus swayed my opinion.” And, as another student responded:

Yes, this course has influenced my understanding of the scientific consensus on climate change. Before, I thought that it was more of a debate on whether climate change exists, but scientists are over 90% in agreement on the human influence in climate change. This should no longer be a debate, but rather a discussion on what can be done.

We also asked our students:

On a scale from 0–100, how worried are you about climate change? Answer between 0 and 100, where 0 = I am not at all worried, 50 = neutral, and 100 = I am very worried (van der Linden et al., 2015).

**Figure 3:**
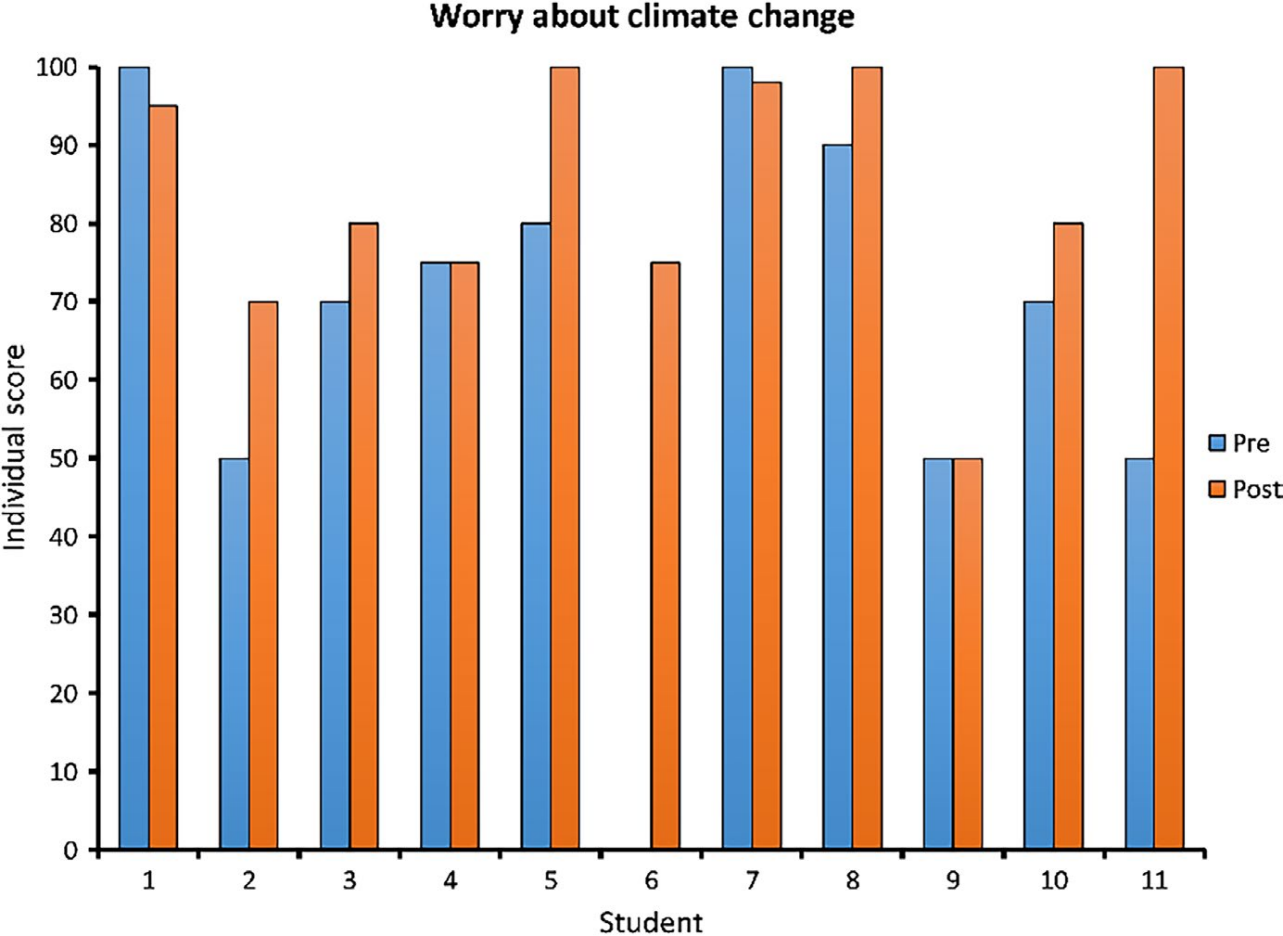
Worry. Scores for students' levels of worry about climate change before and after reading and discussion of a meta‐analysis and a systematic review of current climate science articles. A Wilcoxon signed‐rank test revealed a significant increase in worry about climate change (*Z* = −2.320, *p* = .020)

On the first day of the course, the mean score for worry about climate change was 66.82 out of 100, with a minimum of 0, maximum of 100, and standard deviation of 28.83. After learning about the scientific consensus, the mean score rose to 83.91, with a minimum of 50, maximum of 100, and standard deviation of 16.22. A Wilcoxon signed‐rank test found that the increase in the degree to which students were worried about climate change between the pretest and post‐test was significant (*Z* = −2.320, *p* = .020; Figure 3). This measurement matches students' self‐reporting, as seven out of the 10 students who expressed opinions regarding whether the course had changed their level of worry about climate change answered that it had. Furthermore, all but one of them directly attributed their shift in degree of concern to exposure to the scholarly literature. Representative student statements included:

[This course] has made me more worried about climate change because a large percentage of the public does not understand that humans are the cause of climate change, and that experts highly agree on this.

I am slightly more concerned than I was previously, simply because seeing such a disagreement in the public is frightening to think about.

Yes! This course made me realize the large gap between the general public and the scientific community and has increased my worrying.

Yes, this course definitely influenced how worried I am about climate change, given that most of the public is unaware of the high scientific consensus regarding human‐caused climate change.

Finally we asked our students:

How confident are you in your ability to communicate the degree of scientific consensus on human‐caused climate change to others? Answer between 0 and 100, where 0 = I am not at all confident, 50 = I am somewhat confident, and 100 = I am completely confident.

**Figure 4:**
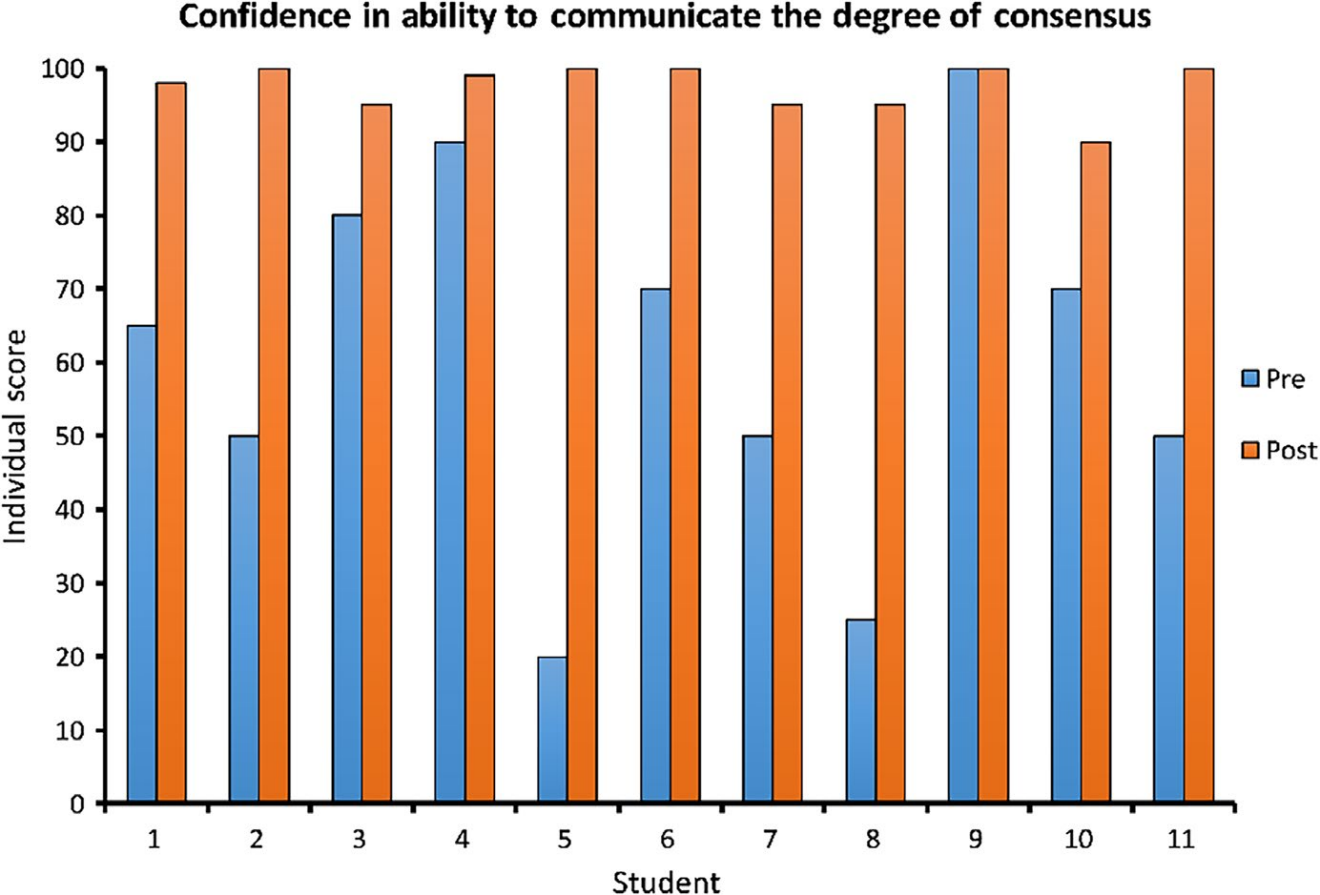
Confidence. Scores for students' confidence in their ability to communicate the scientific consensus on climate change before and after reading and discussion of a meta‐analysis and a systematic review of current climate science articles. A Wilcoxon signed‐rank test revealed a significant increase in confidence in communicating the consensus (*Z* = −2.805, *p* = .005)

At the beginning of the course, the mean score for student confidence in their ability to communicate to others about the consensus was 60.91 out of 100, with a minimum of 20, maximum of 100, and standard deviation of 24.98. After reading and discussion of the meta‐analysis and review regarding the consensus, the mean score increased to 97.45, with a minimum of 90, maximum of 100, and standard deviation of 3.30. A Wilcoxon signed‐rank test found that there was a significant increase in levels of confidence in communicating the consensus between the pretest and post‐test (*Z* = −2.805, *p* = .005; Figure 4).

Qualitative data also suggested an overwhelming improvement in students' confidence in their ability to communicate the degree of scientific agreement on human‐caused climate change. Of the participants who took a direct position on whether the course influenced their confidence (10 of the 11 students in the course), all of them indicated that the course improved their confidence in their ability to communicate the degree of consensus. Representative quotes included:

This course has increased my confidence in communicating the degree of scientific consensus on human‐caused climate change because now I know numbers and percentages that relate to what scientists believe as the cause of global warming. Before this course, I would not have been able to accurately and confidently defend the consensus, but after reading Cook's articles, I can say that I can easily defend the consensus.

Yes, this course has made me more confident in my ability to communicate the degree of scientific consensus on human‐caused climate change to others. Now that I know that the consensus is so high, I have facts and proof to back up my arguments about climate change. Other people think it is all just a hoax, but with this information, I would be able to prove to them that it is, indeed, not a hoax.

and “Yes, I am more comfortable now as I am able to use knowledge of research and statistics regarding consensus among the scientific community to communicate to my peers.”

## 3 DISCUSSION AND CONCLUSION

**Figure 5:**
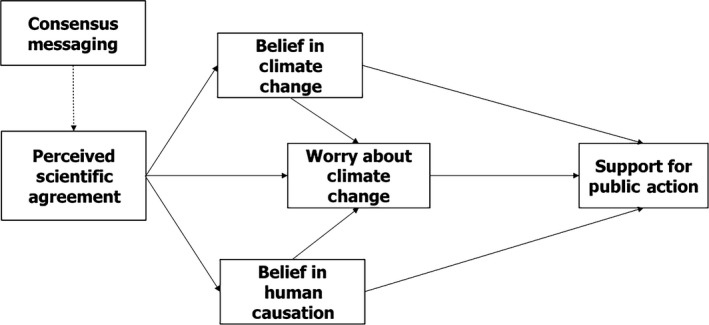
The Gateway Belief Model. (Reproduced with permission from van der Linden et al. (2015))

Our initial results indicate that among our population of climate change accepting biology majors at a research university, our students perceived, on average, that more than a quarter of climate scientists were in doubt regarding anthropogenic climate change. That is, they underestimated the actual consensus by an order of magnitude. Additionally, these upper‐division biology students were, on average, only moderately worried about climate change, and they were not at all confident in their ability to communicate about the state of the scientific consensus around climate change. While ours is, of course, a small sample size without the benefit of a comparison group, this case study suggests that reading and class discussion of the scholarly literature that substantiates the scientific consensus on climate change can be effectively used to improve students' perceptions of the consensus among climate scientists. Perception of such a consensus has been described as a gateway toward acceptance and mobilization to activism (Ding et al., 2011; van der Linden et al., 2015, 2019; McCright et al., 2013) According to the Gateway Belief Model (van der Linden et al., 2015) (see Figure 5), a person's degree of worry about climate change is influenced by their perception of scientific agreement, which is in turn associated with support for action. Our results are in line with this model, as our findings indicate that as students come to understand the scientific consensus, they subsequently become more worried about climate change as well as more able and encouraged to communicate about it to others.

If we wish to dismantle the climate change denial machine, which relies on maintaining an echo chamber shielding deniers from scientific information counter to their prior beliefs and dispositions, we must examine our own echo chambers. We have to begin to understand that, while most students in university‐level biology programs are not likely to be climate change deniers themselves, they may very well not understand the degree of the scientific consensus. Among our students in postsecondary biology programs are those who will become science teachers in K‐12 settings. Although support for teaching about climate change varies locally, there is broad agreement (79%) nationally that “schools should teach about the causes, consequences, and potential solutions to global warming” (Marlon, Howe, Mildenberger, Leiserowitz, & Wang, 2018). However, just as it is a well‐known problem that too many teachers “can't, won't, or don't teach evolution” (Wiles & Branch, 2008), climate change is second only to evolution as a topic that science teachers are likely to avoid due to perceived controversy (Reardon, 2011). Whether or not they will become professional educators, our students are in closer and more consistent contact with their parents and other members of their home communities than their professors are. Hence, they may represent one of our best hopes of breaking into the echo chambers of denial. By helping our students to understand and communicate about the overwhelming consensus among the scientific community, we equip them to bring others closer to where they should be regarding action. In short, while it might seem that taking the time to teach university biology majors about the consensus on climate change may be tantamount to preaching to the choir, it turns out that the choir may well benefit from the sermon.

## CONFLICT OF INTEREST

None declared.

## AUTHOR CONTRUBUTIONS

JDS conceived the main portion of the study and was responsible for quantitative and qualitative data collection and analyses. JDS wrote the first draft of the manuscript, and JDS and JRW contributed to subsequent revisions. JRW directed the project. All authors read and approved the final manuscript.

### Open Research Badge

This article has earned an Open Data Badge for making publicly available the digitally‐shareable data necessary to reproduce the reported results.

## Data Availability

All data supporting results presented herein are reported within this manuscript. No additional data archiving is necessary.
